# Infant Diet Is Associated With BMI Later in Childhood: A Nation‐Wide Mother‐Child Cohort Study in Iceland (ICE‐MCH)

**DOI:** 10.1111/mcn.70165

**Published:** 2026-02-02

**Authors:** Jenny Jonsdottir, Birna Thorisdottir, Kristjana Einarsdottir, Inga Thorsdottir

**Affiliations:** ^1^ Faculty of Food Science and Nutrition, School of Health Sciences University of Iceland Reykjavík Iceland; ^2^ Unit for Nutrition Research, Health Science Institute University of Iceland Reykjavik Iceland; ^3^ The Kids Research Institute Australia Nedlands Western Australia Australia; ^4^ School of Population Health Curtin University Bentley Western Australia Australia; ^5^ Institute for Health Research University of Notre Dame Fremantle Western Australia Australia; ^6^ Department of Nutrition, Exercise and Sports (NEXS), Section for Nutrition and Health University of Copenhagen Copenhagen Denmark

**Keywords:** BMI for age, breastfeeding, child obesity, complementary feeding, dietary index, infant nutrition

## Abstract

Few studies have explored associations between indexes incorporating both breastfeeding and complementary feeding and future risk of overweight/obesity. The aim of this study was to explore associations between a previously developed Infant Diet Score (IDS; higher score reflecting better alignment with breastfeeding and complementary feeding guidelines in the first year of life), and the risk of overweight and/or obesity in childhood. Nutrition and anthropometric data for all children born in Iceland in January 2009 to June 2015 were gathered from national health records. Logistic regression models were used to test associations between IDS and BMI‐for‐age *z*‐scores (WHO standards). Among children for which the IDS could be calculated, anthropometric data was available for 6,335 children at 2.5 years (thereof 7% with overweight/obesity), 2,486 at 4 years (4% with overweight/obesity), 8,946 at 6 years (19% with overweight and 8% obesity) and 5,626 at 9 years (23% with overweight and 15% obesity). Compared to children in the highest IDS quintile, those in quintiles 1 and 2 had higher odds of obesity at 6 years (aOR: 1.42; 95% CI: 1.05–1.93 and aOR: 1.58; 95% CI: 1.18–2.14) and 9 years (aOR: 1.37; 95% CI: 1.02–1.85 and aOR: 1.46; 95% CI: 1.10–1.94). The same applied for IDS quintile 3 in partly, but not fully adjusted models. Associations were inconsistent at 2.5 years and not observed at 4 years. In this national cohort, lower alignment with infant nutrition guidelines was associated with higher risk of obesity at school age.

## Summary

- Infant dietary practices during the first year of life, particularly breastfeeding, play a role in shaping the risk of obesity later in childhood.
- Lower alignment with infant nutrition guidelines during the first year of life was associated with increased odds of obesity at school age, specifically at 6 and 9 years.
- These findings underscore the importance of promoting alignment with established infant nutrition guidelines as a strategy to reduce the risk of childhood obesity. Continued research focusing on obesity in toddlers is also warranted.
- National health records represent a valuable resource for investigating the relationship between infant nutrition and childhood growth and anthropometric outcomes.

## Graphical abstract

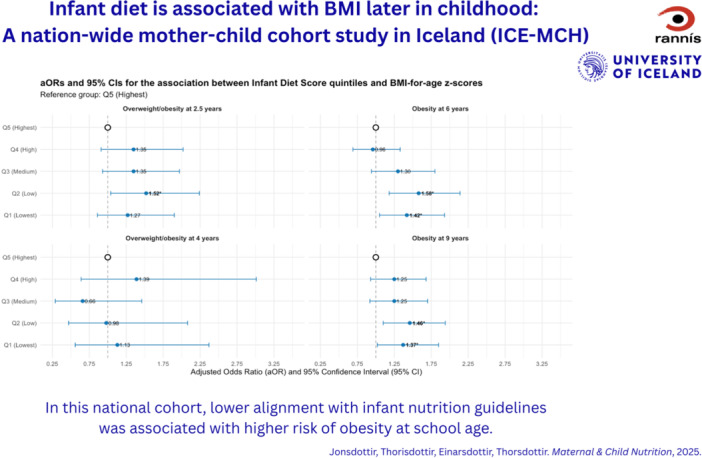

## 1 Introduction

Overweight and obesity is increasing worldwide. Between 1990 and 2022, the global prevalence of obesity more than doubled among adults and quadrupled among school‐aged children and adolescents (Phelps et al. 2024; World Health Organization WHO 2024d). Studies have found that childhood obesity is associated with insulin resistance, impaired glucose tolerance (Holst‐Schumacher et al. 2008), and elevated cardiometabolic risk (Nur Zati Iwani et al. 2023). Also, obesity during childhood can negatively impact quality of life and significantly increase the risk of developing non‐communicable diseases (NCDs) later in life, including several types of cancer and cardiovascular conditions such as hypertension and type 2 diabetes (GBD 2021 Risk Factor Collaborators 2021 Risk Factor Collaborators 2024).

Nutrition during the first year of life undergoes many changes as infants transition from breast milk to family foods. Ensuring proper nutrition during this period is crucial for both immediate and long‐term health at the individual and population levels (Hörnell and Lagström 2024; Nordic Council of Ministers 2023; Scientific Advisory Committee on Nutrition 2018; World Health Organization WHO and the United Nations Children's Fund UNICEF 2023). Composite indexes that assess the extent to which infant dietary practices align with nutritional guidelines offer an innovative methodological approach for investigating the relationship between early‐life nutrition and subsequent growth or health outcomes. However, relatively few studies have developed composite indexes that incorporate both breastfeeding practices and complementary feeding components during the first year of life (Agnihotri et al. 2021; Au et al. 2023; Castro et al. 2022; Golley et al. 2012; Golley et al. 2013; Meyerkort et al. 2012; Ríos et al. 2016) and explore their associations with growth or risk of overweight/obesity (Au et al. 2023; Castro et al. 2022; Golley et al. 2013; Meyerkort et al. 2012; Okubo et al. 2015; Ríos et al. 2016). Among these, most have reported inverse associations between scores reflecting greater adherence to infant nutrition guidelines and measures of higher BMI, overweight, or obesity (Au et al. 2023; Castro et al. 2022; Okubo et al. 2015; Ríos et al. 2016), though some found no significant associations (Golley et al. 2013; Meyerkort et al. 2012). For example, a U.S.‐based study found that a higher Infant Diet Quality Index score, reflecting better adherence to diet recommendations, was associated with lower BMI z‐scores at both 2 and 4 years of age (Au et al. 2023). Similarly, a study from New Zealand identified associations between an Infant Feeding Index and overweight/obesity at 4.5 years (Castro et al. 2022). In a U.K. birth cohort, a Diet Quality Index was linked to measures of adiposity, though not BMI, at 6 years of age (Okubo et al. 2015). Recently, we developed a comprehensive Infant Diet Score (IDS) that integrates six indicators, including both breastfeeding and complementary feeding, to evaluate alignment with infant nutrition guidelines during the first year of life. We found that lower IDS scores, indicating poorer alignment with recommendations, were associated with higher odds of overweight and obesity at 12 and 18 months of age (Jonsdottir et al. 2025).

The present study aims to investigate the associations between infant nutrition and childhood body weight using data from the registry‐based Icelandic Maternal and Child Health Study (ICE‐MCH 2002–2015), which is derived from health records within Iceland's well‐established primary care system. Specifically, we examined the associations between the recently developed IDS and the risk of overweight and/or obesity at 2.5, 4, 6, and 9 years of age.

## 2 Methods

### 2.1 Study Population and Data

**Figure 1:**
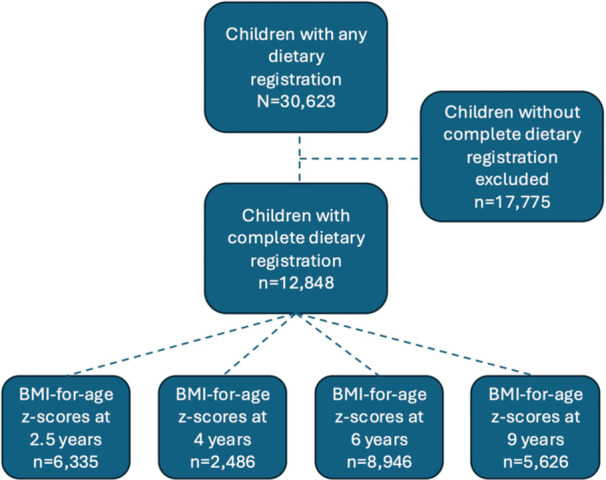
Flow chart of the data selection process for the ICE‐MCH cohort analysis.

We obtained data from the registry‐based Icelandic‐Maternal‐and‐Child‐Health‐Study (ICE‐MCH) on all Icelandic children born during the years 2009–2015. Data was obtained from three nationwide health records: the Icelandic Medical Birth Registry (weight and length at birth, gestational age, parity, singleton birth/multiple births, and mode of delivery), the Saga Maternal and Child Health Database (infant sex, residency, maternal age, maternal employment, pre‐pregnancy weight and height, parental cohabitation, infant nutrition, weight, and length/height up to 4 years) and the Ískrá School Health Care registry (weight and height at 6 and 9 years). The study population included 30,623 children. A total of 17,775 children were excluded due to incomplete dietary registrations from birth to 12 months. The final analyses were conducted on a subgroup of 12,848 children with complete dietary data (representing 42% of the full ICE‐MCH cohort) (Figure 1). As previously published, their birth, maternal and demographic distribution were considered representative of the full cohort (Jonsdottir et al. 2025).

### 2.2 Nutritional Data

In Saga Maternal and Child Health Database, nutritional data were recorded as a binary variable at each health care screenings in infancy at ages 1–5 weeks (median 3 weeks), 6 weeks, 9 weeks, 3 months, 5, 6, 8, 10 and 12 months. These include breastfeeding status (any and exclusive), consumption of infant formula, follow‐on milk or cow's milk, intake of complementary foods (porridge, fish, meat, and fruit/vegetables), and use of vitamin D supplements (vitamin D drops or fish oil). Based on these records covering 0–12 months of age, a composite IDS ranging from 0 to 5 was constructed for children with complete dietary registration from birth until 1 year of age as published earlier (Jonsdottir et al. 2025). IDS was composed of six components representing alignment with infant dietary recommendations (Jonsdottir et al. 2025); duration of EBF and any BF, the age of first introduction of cow's milk and semi‐solids/solids, food group variety from 8 to 12 months and vitamin D supplementation during the first year of life. IDS components were based on similar previously conducted dietary indexes among infants (Au et al. 2023; Castro et al. 2022) but additionally included a vitamin D supplementation score. The structure is summarized in Supporting Information Table 1.

In the study population, the IDS representing alignment with nutrition guidelines in the first year of life, ranged from 0.50 to 4.83. Children were divided into quintiles, with the lowest quintile (Q1) indicating the lowest alignment with current infant nutrition guidelines. The highest quintile (Q5) was used as the reference group and indicates complete alignment with infant nutrition guidelines. The ranges were as follows: quintile 1 (IDS ≤ 2.52), quintile 2 (2.52 < IDS ≤ 3.23), quintile 3 (3.23 < IDS ≤ 3.67), quintile 4 (3.67 < IDS ≤ 4.08), and quintile 5 (IDS > 4.08).

Quintiles of the IDS were obtained across the entire study population (*n* = 12,848) based on the combined dietary information from 0 to 12 months.

### 2.3 Outcomes and Definitions

BMI‐for‐age *z*‐scores (BMI ***z***) were calculated according to WHO Child Growth Standards from 2006 (World Health Organization (World Health Organization WHO 2024b) and were calculated by age‐and‐sex (among children under 5 years) and the WHO References published in 2007 for school‐aged children and adolescents (from 5 to 19 years of age) (World Health Organization (World Health Organization WHO 2024c). The R “Anthro” package was used for children under 5 years of age (< 60 months) (World Health Organization (World Health Organization WHO 2024e) and the R package “AnthroPlus” was used to calculate z‐scores among children from 5 years (> 60 months) (World Health Organization (World Health Organization WHO 2024a). Weight status categories were classified according to the BMI*z* relevant cut‐offs. The cut‐offs values differ between children aged < 60 months and beyond 60 months. Children aged 2.5 and 4 years of age were classified as overweight if their BMI*z* was >+2 and obese if their BMI*z* was >+3. We used overweight and obesity combined (>+2) in analyses among children aged 2.5 and 4 year as has been done in prior studies (Castro et al. 2022; Jonsdottir et al. 2025). For children aged 6 and 9 years, overweight was defined as a *z*‐score >+1 to <+2, and obesity as a *z*‐score >+2. As recommended, children with <−5 or >+5 BMI*z* were excluded from the analyses (World Health Organization WHO 2009; World Health Organization WHO and the United Nations Children's Fund UNICEF 2019). The numbers of children excluded at ages 2.5, 4, 6, and 9 years were 7, 4, 64, and 37, respectively.

### 2.4 Covariates

Confounders used for the analysis were the following: infant birth weight (continuous variable), urban/rural residence (capital, outside capital area), maternal age (continuous variable), pre‐pregnancy BMI (kg/m^2^, continuous variable), employment (employed, student, unemployed/pension/other), and parental cohabitation (yes, no).

### 2.5 Statistical Analysis

Descriptive statistics were conducted to describe the population by BMI*z* and subsequent weight categories at ages 2.5, 4, 6 and 9 years. Means and standard deviations were calculated for continuous variables and counts and proportions were used to describe categorical variables.

Differences in continuous characteristics between groups were assessed using Welch's two‐sample *t*‐test, and differences in categorical characteristics were assessed using Pearson's chi‐squared test. A *p*‐value of < 0.05 was considered statistically significant.

Logistic regression analysis with odds ratios (OR) and 95% confidence intervals (CI) was conducted to evaluate the association between the IDS quintiles (independent variable) and the odds of overweight and obesity (dependent variable) at ages 2.5, 4, 6, and 9 years. Model 1 was unadjusted, model 2 was adjusted for birth weight, and residency, and model 3 was adjusted for birth weight, residency, maternal pre‐pregnancy BMI, age and employment as well as parental cohabitation. Logistic regression was also used to assess the association between IDS six varying components (independent variables) and the odds of overweight and obesity (dependent variables) at the same ages (2.5, 4, 6, and 9 years), both unadjusted (model 1), and adjusted (model 2 and 3). In addition, associations between each individual IDS component score modeled as continuous variables (independent variables) and the odds of obesity (dependent variable) at ages 6 and 9 years were evaluated. ORs and 95% CIs were estimated per one‐unit increase in each component score using unadjusted (model 1) and adjusted models (models 2 and 3).

Mean BMI*z* trajectories from 1 to 9 years of age were plotted by IDS quintile to descriptively illustrate longitudinal patterns in BMI*z* development.

Statistical analysis was conducted using R Statistical Software (version 4.2.3) (R Core Team 2023).

### 2.6 Ethical Approval

The ICE‐MCH study was approved by the Icelandic Data Protection Authority (ref. 2014050799) and the National Bioethics Committee in Iceland (VSN‐14‐078), with later additions. Access and linking of database information in the ICE‐MCH study was approved by the Directorate of Health (no. 14050304). Access to information in the Saga Maternal and Child Health Database was additionally approved by the Primary Health Care of the Capital Area (ref. lA3g/22/845.l) and other health care centers in the country.

## 3 Results

The study population included 51% boys, 93% children born at term, with the mean birth weight and length 3.64 kg (SD 0.53) and 51.2 cm (SD 2.3). Mean maternal age was 29.5 years (SD 5.5) and mean maternal pre‐pregnancy BMI 26.2 kg/m^2^ (SD 5.4). Most mothers were employed (78%), living in the capital area (70%) and with a partner (82%). The percentage of children categorized as having overweight or obesity was 7% at 2.5 years, 4% at 4 years, 27% at 6 years (thereof 8% obese) and 38% at 9 years (thereof 15% obese) (Table 1). Anthropometrics were similar between the whole cohort and the study population of children with information on the IDS.

**Table 1:** Anthropometric characteristics of all children and the sub‐set of participants for which the Infant Diet Score (IDS) was calculated.

	All children (*N* = 30,623)	Children with IDS (*n* = 12,848)	
	Mean ± SD or *N* (%)	Mean ± SD or *N* (%)	*p*‐value^a^
**At 2.5 years**	***N*** = **12,862**	***n*** = **6335**	
BMI‐for‐age *z*‐score	0.60 ± 0.95	0.61 ± 0.94	0.744
Weight category			0.738
Underweight (BMI*z* <−2)	57 (0.4)	26 (0.4)	
Normal weight	11,934 (92.8)	5877 (92.8)	
Overweight (BMI*z* >+2)	767 (6.0)	385 (6.1)	
Obese (BMI*z* >+3)	104 (0.8)	47 (0.7)	
**At 4 years**	***N*** = **7383**	***n*** = **2486**	
BMI‐for‐age *z*‐score	0.48 ± 0.93	0.48 ± 0.91	0.868
Weight category			0.108
Underweight (BMI*z* <−2)	23 (0.3)	6 (0.2)	
Normal weight	6983 (94.6)	2371 (95.4)	
Overweight (BMI*z* >+2)	307 (4.2)	93 (3.7)	
Obese (BMI*z* >+3)	70 (0.9)	16 (0.6)	
**At 6 years**	***N*** = **17,961**	***n*** = **8946**	
BMI‐for‐age *z*‐score	0.44 ± 1.09	0.45 ± 1.08	0.253
Weight category			0.233
Underweight (BMI*z* <−2)	163 (0.9)	70 (0.8)	
Normal weight	13,009 (72.4)	6453 (72.1)	
Overweight (BMI*z* >+1)	3343 (18.6)	1707 (19.1)	
Obese (BMI*z* >+2)	1446 (8.1)	716 (8.0)	
**At 9 years**	***N*** = **12,514**	***n*** = **5626**	
BMI‐for‐age *z*‐score	0.67 ± 1.19	0.69 ± 1.17	**0.036**
Weight category			0.111
Underweight (BMI*z* <−2)	105 (0.8)	37 (0.7)	
Normal weight	7801 (62.3)	3476 (61.8)	
Overweight (BMI*z* >+1)	2804 (22.4)	1294 (23.0)	
Obese (BMI*z* >+2)	1804 (14.4)	819 (14.6)	

*Note: Z*‐scores were calculated by age‐ and sex (WHO Growth Standards/References). Bold values indicate statistical significance (*p* < 0.05).

^a^ Welch two samples *t*‐test for continuous variables; Pearson's chi‐squared test for categorical variables.

Children with IDS in quintiles 1 and 2 had higher odds of obesity at 6 and 9 years, compared with children in the highest IDS quintile (Table 2). The same applied for IDS quintile 3 in unadjusted and partly adjusted, but not fully adjusted models. The fully adjusted model was limited to children with complete information on all covariates and therefore included fewer children. With the exception of IDS quintile 2 and overweight/obesity at 2.5 years, no associations were found between low IDS quintiles and overweight/obesity at 2.5 or 4 years, nor overweight, without obesity, at 6 or 9 years in fully adjusted models.

**Table 2:** ORs and 95% CIs for the association between Infant Diet Score quintiles and BMI‐for‐age *z*‐scores at 2.5 years, 4 years, 6 years and 9 years of age.

		Infant Diet Score quintiles	
	Model 1 (unadjusted)	Model 2 (adjusted)^a^	Model 3 (fully adjusted)^b^
	OR (95% CI)	aOR (95% CI)	aOR (95% CI)
**At 2.5 years**, *N*	6335	6335	4588
BMI*z* > 2*			
Q5 (highest)	*Ref*	*Ref*	*Ref*
Q4 (high)	1.05 (0.74–1.48)	1.09 (0.77–1.55)	1.35 (0.91–2.02)
Q3 (medium)	1.26 (0.92–1.73)	1.27 (0.93–1.76)	1.35 (0.93–1.97)
Q2 (low)	**1.39 (1.02–1.91)**	**1.52 (1.11–2.10)**	**1.52 (1.04–2.24)**
Q1 (lowest)	1.15 (0.83–1.60)	1.28 (0.92–1.79)	1.27 (0.86–1.90)
**At 4 years**, *N*	2486	2486	1753
BMI*z* > 2*			
Q5 (highest)	*Ref*	*Ref*	*Ref*
Q4 (high)	1.18 (0.61–2.29)	1.19 (0.61–2.32)	1.39 (0.64–3.01)
Q3 (medium)	0.93 (0.49–1.77)	0.90 (0.48–1.71)	0.66 (0.29–1.46)
Q2 (low)	1.35 (0.74–2.50)	1.33 (0.73–2.48)	0.98 (0.47–2.08)
Q1 (lowest)	1.30 (0.71–2.43)	1.32 (0.72–2.47)	1.13 (0.56–2.37)
**At 6 years**, *N*	8946	8946	6800
BMI*z* > 1 to < 2**			
Q5 (highest)	*Ref*	*Ref*	*Ref*
Q4 (high)	**1.21 (1.02–1.44)**	**1.22 (1.03–1.45)**	**1.22 (1.01–1.49)**
Q3 (medium)	1.17 (0.98–1.41)	1.18 (0.98–1.42)	1.18 (0.96–1.45)
Q2 (low)	1.08 (0.91–1.28)	1.13 (0.95–1.34)	0.96 (0.78–1.17)
Q1 (lowest)	**1.20 (1.01–1.43)**	**1.27 (1.07–1.51)**	1.08 (0.89–1.33)
BMI*z* > 2***			
Q5 (highest)	*Ref*	*Ref*	*Ref*
Q4 (high)	1.01 (0.76–1.34)	1.00 (0.75–1.32)	0.96 (0.69–1.33)
Q3 (medium)	**1.49 (1.13–1.96)**	**1.46 (1.11–1.93)**	1.30 (0.94–1.80)
Q2 (low)	**1.80 (1.40–2.32)**	**1.84 (1.43–2.38)**	**1.58 (1.18–2.14)**
Q1 (lowest)	**1.74 (1.35–2.26)**	**1.78 (1.38–2.31)**	**1.42 (1.05–1.93)**
**At 9 years**, *N*	5626	5626	4060
BMI*z* > 1 to < 2**			
Q5 (highest)	*Ref*	*Ref*	*Ref*
Q4 (high)	0.86 (0.70–1.06)	0.87 (0.70–1.08)	0.99 (0.79–1.24)
Q3 (medium)	1.11 (0.92–1.35)	1.11 (0.92–1.35)	0.96 (0.75–1.22)
Q2 (low)	0.97 (0.80–1.18)	1.00 (0.82–1.21)	0.91 (0.72–1.14)
Q1 (lowest)	0.96 (0.78–1.17)	1.00 (0.82–1.22)	0.87 (0.69–1.10)
BMI*z* > 2***			
Q5 (highest)	*Ref*	*Ref*	*Ref*
Q4 (high)	1.17 (0.89–1.53)	1.16 (0.89–1.53)	1.25 (0.93–1.68)
Q3 (medium)	**1.32 (1.03–1.69)**	**1.29 (1.01–1.65)**	1.25 (0.92–1.70)
Q2 (low)	**1.65 (1.30–2.11)**	**1.66 (1.30–2.13)**	**1.46 (1.10–1.94)**
Q1 (lowest)	**1.56 (1.22–2.00)**	**1.61 (1.26–2.07)**	**1.37 (1.02–1.85)**

Abbreviations: aOR, adjusted odds ratios; OR, odds ratios; Ref, reference group; 95% CI, 95% confidence interval.

*Note: Z*‐scores were calculated by age‐ and sex. Infant Diet Score quintiles were defined as follows: Q1 (≤ 2.52), Q2 (2.52–3.23), Q3 (3.23–3.67), Q4 (3.67–4.08), and Q5 (> 4.08). Infant Diet Score quintile 5 (Q5) represents highest alignment with nutrition guidelines and quintile 1 (Q1) represents lowest. Bold values indicate statistically significance (*p* < 0.05).

* Indicative of overweight/obesity at 2.5 and 4 years (WHO Multicentre Growth Reference Study Group 2006).

** Indicative of overweight and

*** indicative of obesity at 6 and 9 years (WHO References, 2007).

^a^ Adjusted for birth weight and residency.

^b^ Adjusted for birth weight, residency, pre‐pregnancy BMI, maternal age, maternal employment and parental cohabitation.

Further exploration of the IDS six components is shown in Table 3. Exclusive and any breastfeeding components of the IDS were inversely associated with BMI*z* >+2 at ages 6 and 9 years, with lower scores corresponding to higher odds of obesity. However, the association for exclusive breastfeeding was no longer statistically significant in the fully adjusted model for children aged 9 years.

**Table 3:** ORs and 95% CIs for the association between Infant Diet Score components and BMI‐for‐age *z*‐scores at 6 years and 9 years.

	BMI*z* > 2 at 6 years***			BMI*z* > 2 at 9 years***		
	Model 1 (unadjusted) *n* = 8946	Model 2 (adjusted)^a^ *n* = 8946	Model 3 (fully adjusted)^b^ *n* = 6800	Model 1 (unadjusted) *n* = 5626	Model 2 (adjusted)^a^ *n* = 5626	Model 3 (fully adjusted)^b^ *n* = 4060
Components	OR (95% CI)	aOR (95% CI)	aOR (95% CI)	OR (95% CI)	aOR (95% CI)	aOR (95% CI)
Exclusive breastfeeding score	**0.49 (0.39–0.61)**	**0.54 (0.44–0.67)**	**0.62 (0.47–0.81)**	**0.62 (0.50–0.77)**	**0.60 (0.48–0.74)**	0.79 (0.61–1.04)
Any breastfeeding score	**0.56 (0.46–0.69)**	**0.47 (0.38–0.59)**	**0.73 (0.57–0.94)**	**0.59 (0.48–0.72)**	**0.56 (0.46–0.69)**	**0.70 (0.55–0.90)**
Age of first introduction of cow's milk score	0.60 (0.31–0.120)	0.79 (0.41–1.60)	0.90 (0.40–2.15)	0.60 (0.35–1.06)	0.80 (0.46–1.42)	0.96 (0.48–2.00)
Age of first introduction of semi‐solids/solids score	0.77 (0.58–1.02)	0.76 (0.57–1.01)	**0.68 (0.49–0.96)**	1.06 (0.79–1.43)	1.04 (0.78–1.41)	1.07 (0.75–1.53)
Number of food groups score	**1.72 (1.23–2.41)**	**1.55 (1.10–2.17)**	1.28 (0.86–1.92)	**1.49 (1.07–2.08)**	1.34 (0.96–1.87)	1.14 (0.76–1.70)
Vitamin D supplement score	**0.56 (0.33–0.97)**	0.65 (0.38–0.13)	0.67 (0.35–1.28)	0.78 (0.35–1.77)	0.97 (0.44–2.24)	0.75 (0.29–2.05)

Abbreviations: aOR, adjusted odds ratios; OR, odds ratios; Ref, reference group; 95% CI, 95% confidence interval.

*Note:* Odds ratios represent the change in odds of obesity per one‐unit increase in the respective IDS component score. Component score ranges were: exclusive breastfeeding (0–1), any breastfeeding (0–1), age of first introduction of cow's milk (0–0.5), age of first introduction of semi‐solids/solids (0–1), number of food groups (0–1), and vitamin D supplementation (0–0.5). Higher component scores reflect better alignment with infant feeding guidelines. *Z*‐scores were calculated by age and sex. Bold values indicate statistically significance (*p* < 0.05).

*** Indicative of obesity at 6 and 9 years (WHO References, 2007).

^a^ Adjusted for birth weight and residency.

^b^ Adjusted for birth weight, residency, pre‐pregnancy BMI, maternal age, maternal employment and parental cohabitation.

**Figure 2:**
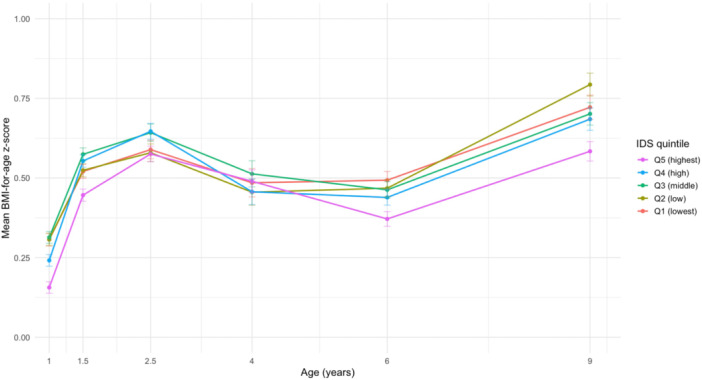
BMI‐for‐age *z*‐score trajectories from 1 to 9 years by Infant Diet Score quintiles.

Across ages 1–9 years, mean BMI*z* trajectories differed by IDS quintile, with differences becoming more apparent at ages 6 and 9 years (Figure 2).

## 4 Discussion

Dietary indexes are commonly used in adult nutrition research (Asghari et al. 2017; Shams‐White et al. 2023), and they offer promising and innovative opportunities to adopt more holistic approaches in the study of infant nutrition (Dalwood et al. 2020). The IDS used in the present study is particularly valuable because it incorporates both breastfeeding practices (exclusive and any breastfeeding) and complementary feeding practices. This includes the timing of introduction of semi‐solid/solid foods and cow's milk, dietary diversity, and additionally vitamin D supplementation. Studies of infant diet indexes possible associations with childhood growth are scarce (Au et al. 2023; Castro et al. 2022; Golley et al. 2013; Meyerkort et al. 2012; Ríos et al. 2016), and findings to date have been mixed (Golley et al. 2013; Meyerkort et al. 2012).

Our results show that lower alignment with infant nutrition guidelines is associated with increased odds of obesity at 6 and 9 years of age. In fully adjusted models, children in the two lowest quintiles had 37%–58% higher odds of obesity compared to those with higher alignment. No significant associations were observed with overweight alone (excluding obesity) at these same ages after adjusting for covariates. This distinction between overweight and obesity has also been reported in previous studies (Shields et al. 2006). These findings suggest that infant dietary practices may be more strongly associated with more severe forms of excess weight, although the mechanisms driving this distinction remain unclear.

At 2.5 years of age, children in the second‐lowest quintile of the IDS had increased odds of overweight and/or obesity, whereas no association was observed for those in the lowest quintile. At 4 years, no significant associations were found between IDS and overweight and/or obesity. These findings differ from those reported in a U.S. study, which found that lower infant diet quality scores were associated with higher BMI z‐scores at comparable ages—2 and 4 years (Au et al. 2023). However, the US study did not find an association between lower index scores and BMI at 3 years of age. Our previous research identified associations between the IDS and overweight/obesity at 12 and 18 months of age (Jonsdottir et al. 2025). It seems crucial to further advance our understanding of how nutrition during infancy can possibly influence the risk for overweight/obesity as part of a prevention strategy. This is particularly important for age groups in which obesity prevalence is increasing, such as at 6 and 9 years. The inconsistent patterns of association observed across different child age groups and studies underscore the need for further research. Such investigations are critical to clarifying the long‐term impact of early dietary practices on child growth and weight outcomes.

Globally, several studies and systematic reviews have reported associations between breastfeeding and a reduced risk of overweight and obesity (Horta et al. 2023; Ip et al. 2009; Qiao et al. 2020; Scientific Advisory Committee on Nutrition 2018; Victora et al. 2016; Yan et al. 2014), as well as between greater adherence to infant nutrition guidelines and lower risk of excess weight (Au et al. 2023; Castro et al. 2022). However, the evidence across the literature remains mixed. Some studies have found weak or non‐significant associations between breastfeeding practices and later obesity outcomes (Kramer and Kakuma 2012; Morgen et al. 2018; Owen et al. 2005), highlighting the complexity of this relationship and the potential influence of other contributing factors.

The present study contributes to the growing body of evidence on infant nutrition by examining associations between infant dietary practices and obesity risk at 6 and 9 years of age. In our earlier research, four out of six components of the IDS were associated with lower odds of elevated body weight at 1.5 years (Jonsdottir et al. 2025). In the current analysis, two of these components, i.e. EBF and BF, were similarly associated with reduced odds of obesity at 6 and 9 years. These findings reinforce the significance of adhering to breastfeeding recommendations to support healthy weight trajectories and growth patterns in childhood, consistent with previous studies (Horta et al. 2023; Qiao et al. 2020; Rito et al. 2019; Rzehak et al. 2017; Victora et al. 2016). Slower growth velocity of breastfed infants in late infancy is not necessarily a concern; rather, it may protect against higher BMI peaks in later childhood (Rzehak et al. 2017). The long‐term benefits of sustained breastfeeding have been well documented—not only in promoting healthy growth and reducing obesity risk, but also in lowering the incidence of gastrointestinal infections and type 2 diabetes (Frank et al. 2019; Horta et al. 2023; Kramer and Kakuma 2012; Victora et al. 2016). Notably, most prior research has focused on early childhood outcomes. This study extends the existing literature by exploring the influence of infant dietary practices on obesity risk into later childhood, up to 9 years of age.

Obesity is a complex, multifactorial health condition associated with an increased risk of NCDs and reduced quality of life (GBD 2021 Risk Factor Collaborators 2021 Risk Factor Collaborators 2024; Lundgaard and Lourenço 2024). The World Health Organization Growth Standards, developed from data on healthy children raised in optimal conditions, are intended for global application. However, their suitability has been a subject of debate, particularly with respect to assessing nutritional status across diverse populations (Borghi et al. 2024). Growth references differ in their underlying data sources and cut‐off criteria (Centers for Disease Control and Prevention CDC 2024; Cole and Lobstein 2012; Rolland‐Cachera 2011; World Health Organization WHO 2024b; World Health Organization WHO 2024c), which can result in varying prevalence estimates for overweight and obesity depending on the reference applied. In clinical practice, Icelandic healthcare providers use either the Swedish growth reference charts (Wikland et al. 2002) or the international cut‐offs developed by Cole and colleagues (Cole 2000). The prevalence of obesity among 6‐year‐olds observed in the present study was 8%, a higher figure than the national report on school‐aged children in Iceland (Development Centre for Primary Healthcare in Iceland DCPHI 2020), where the prevalence of obesity among 6‐year‐olds was reported 6.4% in girls and 6.5% in boys. This difference is expected given the use of WHO Growth Standards in the present study, which are based on optimal growth trajectories (Centers for Disease Control and Prevention CDC 2024; World Health Organization WHO 2024b; World Health Organization WHO 2024c). Despite methodological differences among growth references, growth charts remain essential tools for tracking growth patterns and identifying deviations that may signal underlying health, nutritional, or lifestyle issues. Nonetheless, establishing a global consensus on the most appropriate growth references would improve the comparability of prevalence estimates across populations and enhance the utility of growth data in both clinical and research settings.

### 4.1 Strength and Limitations

A major strength of this study is the use of a large, nationally representative dataset comprising approximately 13,000 children with complete dietary records. This comprehensive dataset enabled the application of the IDS, a novel metric specifically developed and derived from the same nationwide health records used in this analysis. To our knowledge, ours is the first national cohort study to report significant associations between a composite index of infant diet and BMI, reaching through to 9 years of age.

However, some children were excluded due to incomplete data, underscoring the need for consistent and comprehensive data collection within healthcare systems to support high‐quality research in nutritional and health care epidemiology. This limitation was particularly evident for the 4‐year age group, where anthropometric data were available for only 2486 children, fewer than in the other three age groups, potentially affecting the reliability of findings for this age. Nonetheless, the association between the IDS and overweight at 2.5 years was also weaker than those observed at school age, suggesting that the weaker associations at these younger ages may reflect true differences rather than data limitations alone. The reduced sample size at the age of 4 years seems to be attributable to lower attendance at routine health visits during this period.

Finally, the observed increase in obesity prevalence from age 4–6, and further to 9 years, suggests that factors beyond early‐life nutrition may contribute to the development of obesity. While this study adjusted for key maternal and birth‐related variables, additional research is warranted to investigate the influence of environmental and behavioral factors later in childhood.

## 5 Conclusion

In this nationwide cohort, lower alignment with infant nutrition guidelines was associated with an increased likelihood of obesity at school age, specifically at 6 and 9 years. Breastfeeding‐related components of the IDS were particularly associated with these outcomes. Additional research is needed to better understand growth trajectories in toddlers and children up to 4 years of age.

This study contributes to the growing body of evidence linking infant dietary practices with later obesity risk. Promoting alignment with infant nutrition guidelines during the first year of life supports primary prevention of childhood obesity. Public health strategies should prioritize improving diet quality in infancy, including through targeted education and support for parents and caregivers within the healthcare system.

## Author Contributions

J.J., B.T. and I.T. designed research. J.J. conducted research. J.J., B.T., K.E. and I.T. analyzed data. J.J. performed statistical analysis. J.J., B.T. and I.T. wrote paper. I.T. had primary responsibility for final content. All authors read and approved the final manuscript. Data described in the manuscript, code book, and analytic code will not be made available because they are part of an ongoing study.

## Conflicts of Interest

J.J., B.T., K.E., I.T. disclose no conflict of interest.

## Supporting information


**Supplemental Table 1:** The components and scoring of the Infant Diet Score (IDS) by age, for infants born in Iceland during 2009‐2015 (*n*=12,848).

## Data Availability

The data that support the findings of this study are available on request from the corresponding author. The data are not publicly available due to privacy or ethical restrictions.
