# Supplementary material for: Infant Diet Is Associated With BMI Later in Childhood: A Nation‐Wide Mother‐Child Cohort Study in Iceland (ICE‐MCH)
Source: Matern Child Nutr. 2026 Feb 2;22(1):e70165. doi: 10.1111/mcn.70165 (PMC12863415; doi:10.1111/mcn.70165)
Supplement: Supplementary file 1 — Supplemental Table 1: The components and scoring of the Infant Diet Score (IDS) by age, for infants born in Iceland during 2009‐2015 (n=12,848). [file MCN-22-e70165-s001.docx]

**Supplement for: Infant diet is associated with BMI later in childhood: A nation-wide mother-child cohort study in Iceland (ICE-MCH)**

**Supplemental Table 1.** The components and scoring of the Infant Diet Score (IDS) by age, for infants born in Iceland during 2009-2015 (n=12,848).

|  | *I* | *II* | *III* | *IV* | *V* | | | | *VI* |  |
| --- | --- | --- | --- | --- | --- | --- | --- | --- | --- | --- |
|  | Exclusive breastfeeding  score^1^ | Any breastfeeding  score^1^ | Age of first introduction of cow’s milk  score^2^ | Age of first introduction of semi-solids/solids  score^1^ | Number of food groups  Score^1, 3^ | | | | Vitamin D supplement  Score^2^ | Infant Diet Score^4^ |
|  |  |  |  |  | *1* | *2* | *3* | *4* |  |  |
| Age |  |  |  |  |  |  |  |  |  |  |
| 2-3 wks | 0.0333 | 0.0167 | 0.0000 | NA | NA | NA | NA | NA | 0.2500 | The sum of  components  I – VI |
| 6 wks | 0.2300 | 0.1150 | 0.0000 | 0.0000 | NA | NA | NA | NA |  |  |
| 9 wks | 0.3450 | 0.1725 | 0.0000 | 0.0000 | NA | NA | NA | NA |  |  |
| 3 mo | 0.5000 | 0.2500 | 0.0000 | 0.2500 | NA | NA | NA | NA |  |  |
| 5 mo | 0.8333 | 0.4167 | 0.0000 | 0.7500 | NA | NA | NA | NA | 0.2500 |  |
| 6 mo | 1.0000 | 0.5000 | 0.0000 | 1.0000 | NA | NA | NA | NA |  |  |
| 8 mo | NA | 0.6667 | 0.0000 | 0.5000 | 0.2500 | 0.5000 | 0.7500 | 1.0000 |  |  |
| 10 mo | NA | 0.8333 | 0.1667 | 0.0000 | 0.1250 | 0.2500 | 0.3750 | 0.5000 |  |  |
| 12 mo | NA | 1.0000 | 0.5000 | 0.0000 | 0.0625 | 0.1250 | 0.1875 | 0.2500 |  |  |
| None/never | 0.0000 | 0.0000 | 0.5000 | 0.0000 | 0.0000 | | | | 0.0000 |  |

^1^Min: 0.0000; Max: 1.0000.
^2^Min: 0.0000; Max: 0.5000.
^3^Number of food groups score contains four food groups: porridges, vegetables/fruits, meat, fish.
^4^Min: 0.0000; Max: 5.0000.
